# Deep Dive Into Gaps and Barriers to Implementation of Antimicrobial Stewardship Programs in Hospitals in Latin America

**DOI:** 10.1093/cid/ciad184

**Published:** 2023-07-05

**Authors:** Valeria Fabre, Clara Secaira, Sara E Cosgrove, Fernanda C Lessa, Twisha S Patel, Andrea Alvarado Alvarez, Lucy Marleni Anchiraico, Maria del Carmen Bangher, Maria Fernanda Barberis, Maria Sol Burokas, Ximena Castañeda, Angel M Colque, Gabriela De Ascencao, Clara Esquivel, Cecilia Ezcurra, Leandro Abel Falleroni, Natalia Frassone, Maria Isabel Garzón, Carlos Gomez, José Anel Gonzalez, Daniela Hernandez, Diego Laplume, César Guillermo Lemir, Herberth Maldonado Briones, Mario Melgar, Florencia Mesplet, Guadalupe Martinez, Carlos Morales Pertuz, Cristina Moreno, Corina Nemirovsky, Yanina Nuccetelli, Belén Palacio, Nancy Sandoval, Hernan Vergara, Hugo Videla, Silvina Villamandos, Olmedo Villareal, Alejandra Viteri, Rodolfo Quiros

**Affiliations:** Department of Medicine, Division of Infectious Diseases, Johns Hopkins University School of Medicine, Baltimore, Maryland, USA; Department of Anthropology and Sociology, Universidad del Valle, Guatemala City, Guatemala; Department of Medicine, Division of Infectious Diseases, Johns Hopkins University School of Medicine, Baltimore, Maryland, USA; International Infection Control Program, Division of Healthcare Quality Promotion, National Center for Emerging and Zoonotic Infectious Diseases, Centers for Disease Control and Prevention, Atlanta, Georgia, USA; International Infection Control Program, Division of Healthcare Quality Promotion, National Center for Emerging and Zoonotic Infectious Diseases, Centers for Disease Control and Prevention, Atlanta, Georgia, USA; Unidad de Cirugía Cardiovascular de Guatemala, Guatemala City, Guatemala; Clínica Universitaria Privada Reina Fabiola, Cordoba, Argentina; Instituto de Cardiología de Corrientes Juana Francisca Cabral, Corrientes, Argentina; Hospital Nacional Profesor Alejandro Posadas, El Palomar, Argentina; Hospital Italiano de Buenos Aires, Buenos Aires, Argentina; Clínica De La Mujer, Bogota, Colombia; Hospital Complejo Medico Churruca Visca, Buenos Aires, Argentina; Hospital San Benito, Peten, Guatemala; Hospital Alemán, Buenos Aires, Argentina; Hospital Provincial de Rosario, Rosario, Argentina; Hospital San Benito, Peten, Guatemala; Unidad de Cirugía Cardiovascular de Guatemala, Guatemala City, Guatemala; Hospital Privado Universitario de Córdoba, Cordoba, Argentina; Clínica De La Mujer, Bogota, Colombia; Hospital Militar Central San José Centro, Bogota, Colombia; Hospital Irma Lourdes Tzanetatos, Panama, Panamá; Hospital Privado Universitario de Córdoba, Cordoba, Argentina; Hospital Nacional Profesor Alejandro Posadas, El Palomar, Argentina; Hospital San Bernardo, Salta, Argentina; Unidad de Cirugía Cardiovascular de Guatemala, Guatemala City, Guatemala; Hospital Roosevelt, Guatemala, Guatemala; Hospital Cesar Milstein, Buenos Aires, Argentina; Hospital San Bernardo, Salta, Argentina; Hospital Del Tunal, Bogota, Colombia; Hospital Metropolitano, Quito, Ecuador; Hospital Italiano de Buenos Aires, Buenos Aires, Argentina; Instituto de Diagnostico, La Plata, Argentina; Sanatorio Allende Nueva Córdoba, Cordoba, Argentina; Hospital Roosevelt, Guatemala, Guatemala; Hospital Militar Central San José Centro, Bogota, Colombia; Instituto de Diagnostico, La Plata, Argentina; Instituto de Cardiología de Corrientes Juana Francisca Cabral, Corrientes, Argentina; Hospital Irma Lourdes Tzanetatos, Panama, Panamá; Hospital Cesar Milstein, Buenos Aires, Argentina; PROAnet Project Lead and Sanatorio Las Lomas, Buenos Aires, Argentina

**Keywords:** antibiotic stewardship, Latin America

## Abstract

**Background:**

Antimicrobial resistance has worsened in Latin America. There is an urgent need to understand the development of antimicrobial stewardship programs (ASPs) and the barriers to implementing effective ASPs in light of limited national action plans or policies to promote ASPs in the region.

**Methods:**

We performed a descriptive mixed-methods study of ASPs in 5 Latin American countries in March-July 2022. An electronic questionnaire with an associated scoring system (hospital ASP self-assessment) was used, and ASP development was classified based on the scores (inadequate, 0–25; basic, 26–50; intermediate, 51–75; or advanced, 76–100). Interviews among healthcare workers (HCWs) involved in antimicrobial stewardship (AS) inquired about behavioral and organizational factors that influence AS activities. Interview data were coded into themes. Results from the ASP self-assessment and interviews were integrated to create an explanatory framework.

**Results:**

Twenty hospitals completed the self-assessment, and 46 AS stakeholders from these hospitals were interviewed. ASP development was inadequate/basic in 35% of hospitals, intermediate in 50%, and advanced in 15%. For-profit hospitals had higher scores than not-for-profit hospitals. Interview data validated the self-assessment findings and provided further insight into ASP implementation challenges, which included limited formal hospital leadership support, inadequate staffing and tools to perform AS work more efficiently, limited awareness of AS principles by HCWs, and limited training opportunities.

**Conclusions:**

We identified several barriers to ASP development in Latin America, suggesting the need to create accurate business cases for ASPs to obtain the necessary funding for their effective implementation and sustainability.

## Teaser

Using a mixed-methods study, we evaluated barriers to implementing antimicrobial stewardship programs (ASPs) in Latin American hospitals. Priorities include improving AS awareness among hospital leaders and prescribers, formal hospital/public health authorities' support for ASPs, better resource allocation, and improved training opportunities.

Implementation of antimicrobial stewardship programs (ASPs) in healthcare is essential to antimicrobial resistance (AMR) prevention and control efforts. Several cross-sectional surveys that evaluated the development of ASPs in Latin American hospitals found that several core elements of ASPs, such as dedicated personnel and pharmacy expertise, are lacking [1]. Furthermore, most countries in Latin America reported not having implemented a national action plan to combat AMR according to a recent survey [2], and few have conducted national assessments of antibiotic use [3, 4]. There is an urgent need to understand the current challenges to implementing antimicrobial stewardship (AS) activities in the region, including behavioral and organizational factors that have not been well addressed through prior evaluations. To address this gap, we conducted a mixed-methods evaluation of 20 hospital ASPs in 5 Latin American countries.

## METHODS

### Study Design

This descriptive mixed-methods study involved a cross-sectional evaluation of hospital ASPs using a previously validated electronic ASP self-assessment tool [5] (Supplementary Material, ASP Self-Assessment) completed by AS teams that rated the type and quality of AS activities in their hospitals followed by semistructured interviews with those leading ASPs. While the ASP self-assessment collected systematic data across different themes, the interviews provided an opportunity for AS stakeholders to express their first-hand experiences regarding barriers to implementing ASPs. We integrated the results from the interviews and self-assessment to provide an explanatory framework.

The Johns Hopkins Medical Institutions Institutional Review Board (IRB) as well as local IRBs at participating hospitals approved the study.

### Study Participants

Twenty hospitals from Panama, Guatemala, Ecuador, Colombia, and Argentina were recruited for participation through a regional network (PROAnet) in March 2022. Healthcare workers (HCWs) directly involved in AS activities (infectious disease [ID] physicians, microbiologists, and pharmacists) at these institutions were invited to participate in semistructured interviews.

### Data Collection

The ASP self-assessment was electronically delivered to each site (1 per hospital). The survey included 94 questions organized in 5 domains (1, leadership support and accountability; 2, clinical guidelines; 3, strategies to optimize antimicrobial use; 4, monitoring and reporting; 5, education and training and patient safety), with each question including a graded response (meets criteria, partially meets criteria, does not meet criteria, and, in some, not applicable). Each domain could earn up to 100 points, and the total score represented the mean of the sum of all domains. Based on the total score, ASP development was categorized as inadequate (0–25), basic (26–50), intermediate (51–75), and advanced (76–100).

Semistructured interviews were conducted in Spanish by a native Spanish-speaking medical anthropologist (C. S.) online using a videoconference program in March 2022–July 2022. The research team developed the initial interview guide according to the Systems Engineering Initiative for Patient Safety (SEIPS) framework [6], which investigates behavioral and systematic components of healthcare practices and has been previously used to identify barriers and facilitators to infection prevention and AS programs [7, 8] (Supplementary Material, Interview Guide). Interviews lasted between 45 and 60 minutes.

### Statistical Analyses

Results from the self-assessment were analyzed using descriptive statistics. We evaluated results overall and by hospital type (for-profit and not-for-profit). Interview recordings were transcribed and analyzed in the original language. Two raters independently coded selected transcripts to develop a preliminary coding dictionary that was refined as more transcripts were analyzed. Directed content analysis of the interview transcripts was performed focusing on barriers and facilitators of ASP implementation guided by the SEIPS model [6]. Quotations included in the manuscript were translated into English by 2 bilingual authors.

## RESULTS

Twelve not-for-profit and 8 for-profit hospitals completed the ASP self-assessment (Table 1). Thirty-five percent (7 of 20) of hospitals scored 0–50 (inadequate/basic), 50% (10 of 20) 51–76 (intermediate), and 15% (3 of 20) 76–100 (advanced). For-profit hospitals had a numerically higher median overall score compared with not-for-profit hospitals (median score, 60; interquartile range [IQR], 56–69 vs median score, 50; IQR, 38–67, respectively; non-significant). The areas with the lowest scores included human resources, education and training, syndrome-specific guidelines, and monitoring of process measures (Table 2, Supplementary Figure).

**Table1.** Characteristics and Antimicrobial Stewardship Self-Assessment Scores for Participating Hospitals Overall and by Hospital Type

Hospital Characteristic	Overall, N = 20	Not-for-Profit, n = 12	For-Profit, n = 8
Country, %			
Argentina	12	6 (50)	6 (75)
Colombia	3	2 (17)	1 (12.5)
Ecuador	1	…	1 (12.5)
Guatemala	3	3 (25)	…
Panama	1	1 (8)	…
Mean no. of beds, range	268 (45–1000)	302 (45–650)	217 (51–1000)
Medical school affiliation, %	18 (90)	12 (100)	6 (75)
Overall self-assessment score, %			
Inadequate/basic	7 (35)	6 (50)	1 (12.5)
Intermediate	10 (50)	4 (33)	6 (75)
Advanced	3 (15)	2 (17)	1 (12.5)

**Table 2.** Answers to the Antimicrobial Stewardship Program Self-Assessment by Hospital Type

Item Evaluated	For-Profit Hospitals N = 8 (%)	Not-for-Profit Hospitals N = 12 (%)
ASP structure and resources		
There is an official document approving the ASP	4 (50)	6 (50)
The official document includes a designated individual responsible for the ASP	4 (50)	2 (17)
There is a specific budget for AS activities including salary support	0	1 (8)
There are annual AS goals and a strategic plan to achieve the goals of the ASP	8 (100)	8 (66)
There is a specific AS committee	5 (63)	8 (66)
The AS committee has authority	1 (13)	3 (35)
The AS committee meets at least quarterly	1 (13)	1 (8)
The AS committee collaborates with other committees	5 (63)	6 (50)
Physicians have dedicated time for AS activities	1 (13)	3 (25)
Pharmacist have dedicated time for AS activities	0	1 (8)
Physicians from other groups participate in AS meetings	6 (75)	4 (35)
Bedside nurses participate in AS meetings	1 (13)	6 (50)
IT resources		
IT assists with data extraction and reporting (eg, antimicrobial consumption data, patient days)	3 (38)	3 (25)
The hospital has digitized medical records	7 (88)	7 (59)
Microbiology		
The hospital has access to a microbiology laboratory	8 (100)	12 (100)
The microbiology laboratory has technology to identify the most relevant resistance mechanisms	8 (100)	11 (92)
The microbiology laboratory has implemented rapid diagnostic testing	7 (88)	10 (82)
The microbiology laboratory performs selective or cascading susceptibility reporting	8 (100)	10 (82)
The microbiology laboratory reports culture results in a timely manner	8 (100)	12 (100)
The microbiology laboratory disseminates annual antibiograms	7 (88)	8 (66)
Treatment guidelines		
Guidelines are developed and adapted by consensus with multidisciplinary teams	8 (100)	10 (83)
Guidelines are adapted based on local epidemiology and sensitivity patterns	8 (100)	10 (83)
Treatment guidelines include recommendations on the duration of antimicrobial therapy	8 (100)	9 (75)
Treatment guidelines include therapeutic alternatives to allergy to beta-lactams	8 (100)	9 (75)
Community-acquired pneumonia	7 (88)	9 (75)
Hospital-acquired pneumonia including ventilation-associated pneumonia	8 (100)	9 (75)
Urinary tract infections	8 (100)	9 (75)
Skin and soft tissue infections	6 (75)	8 (66)
Intraabdominal infections	5 (63)	7 (58)
Sepsis of unknown source	4 (50)	4 (33)
Surgical prophylaxis	8 (100)	11 (92)
Infections in immunocompromised hosts	7 (88)	8 (66)
Multidrug-resistant organism infections	7 (88)	6 (50)
Pharmacy		
ASP participates in decisions regarding inclusion/exclusion of antimicrobials in the hospital formulary	7 (88)	8 (66)
The hospital has regular access to new antimicrobials	8 (100)	10 (83)
AS interventions		
ASP regularly performs post-prescription review and feedback at 48–72 h	5 (63)	4 (33)
Certain antimicrobials require preauthorization for at least certain areas	6 (75)	9 (75)
ASP conducts handshake stewardship	7 (88)	11 (92)
The hospital has a therapeutic drug monitoring program	8 (100)	9 (75)
The hospital has implemented “auto-stops”	4 (50)	4 (33)
There is a process in place to regularly alert of duplicate therapy (eg, duplicate anerobic coverage)	0	0
Pharmacists regularly participate in antimicrobial dose adjustments	4 (50)	2 (17)
Monitoring and reporting		
There is an established system for routine and ad hoc data collection and analysis for AU/AMR data	1 (13)	4 (33)
The ASP monitors antimicrobial consumption data	7 (88)	10 (83)
ASP monitors adherence to clinical practice guidelines	4 (50)	9 (75)
ASP monitors the adherence to specific interventions implemented	3 (38)	5 (42)
The hospital monitors rates of multidrug-resistant organisms	6 (75)	5 (42)
The hospital monitors *Clostridioides difficile* infection rates	6 (75)	4 (50)
The hospital monitors clinical indicators such as mortality, length of stay, readmission	7 (88)	7 (58)
Education and training		
The AS team has access to training on implementation and evaluation of ASPs	8 (100)	11 (92)
Healthcare workers receive education on AS principles upon hiring on AS principles	3 (38)	4 (33)
Healthcare workers receive education on AS principles	4 (50)	8 (66)
Nurses are trained on antimicrobial use such as drug stability	7 (88)	9 (75)
Patients and/or their families are educated on antimicrobials at discharge	4 (50)	4 (33)
The ASP conducts annual awareness campaigns on the responsible use of AU/AMR	1 (13)	4 (33)
Work climate		
The hospital implements strategies to promote teamwork	4 (50)	3 (25)
	5 (63)	2 (17)
The hospital has an anonymous reporting system to report adverse events	7 (88)	6 (50)

Abbreviations: AS, antimicrobial stewardship; ASP, antimicrobial stewardship program; AU, antibiotic use; IT, information technology.

Of 51 HCWs invited, 46 participated in the interviews (19 physicians, 16 microbiologists, and 11 pharmacists). Their years of experience ranged from 1 to 40 years. The following major themes emerged from integrating results from the self-assessment and interviews: organization and leadership support, behavioral determinants, education and training, tasks, tools and information technology (IT), and external environment (Table 3).

**Organization and leadership support**

**Table 3.** Barriers to and Facilitators of Antimicrobial Stewardship Programs as Perceived by Participants Using the Systems Engineering Initiative for Patient Safety Framework

	Barrier	Facilitator
Organization	Lack of formal leadership support	Hospital accreditation
	Lack of a designated ASP leader	Designated ASP leader
	Lack of dedicated time for ASP	Regular meetings to discuss AS related topics
	Lack of specific ASP goals	Close collaboration with Microbiology
	Inadequate pharmacy and microbiology staffing	Type of hospital (size, type of administration)
	Excessive workload	External audits with feedback
	Compensation model/suboptimal salaries	Hospital leadership engages in AS activities (hospital director is member of AS committee, supports discussion around antibiotic practice changes)
	Frequent staff turnover	
	Hierarchical relationships	
	Limited understanding by hospital leaders of relevance/role of ASP	
	Limited hospital budgets	
	Work climate	
Individuals	Low adherence to guidelines	Multidisciplinary work
	Nonevidence-based antibiotic practices	Time in the job
	Limited role of pharmacists in clinical decision-making	Empowering pharmacists (participation in rounds,
	Limited awareness of local antimicrobial resistance data	
	Limited adoption of AS principles	
	Fear of loss of prescriber autonomy	
	Hierarchical relationships	
Tasks	Inefficient processes for approval of restricted antimicrobials (too cumbersome, frontline providers find loopholes)	Integrating frontline providers in AS activities
	Microbiology laboratory has limited hours	Build ASP within infection prevention and control program
	Inefficiency in communication of microbiology results	Daily rounds
	Lack of integration with infection prevention and microbiology	
	Inability to monitor antibiotic use data on a regular basis	
	Limited role of pharmacist in antimicrobial management	
	Limited training in quality improvement implementation	
	Cost of medication	
Tools and IT	Lack of IT support	Training in quality improvement/patient safety
	Lack of computers	Training in how to change behavior
	Lack of software for microbiology results	Electronic prescriptions
	Limited number of treatment guidelines	Robust EMR to allow efficient tracking of antimicrobial resistance/antibiotic use data
	Limited opportunities for AS training	Microbiology data integrated in EMR
	Lack of electronic prescriptions	
	Data fragmentation	
External environment	Lack of policies that facilitate implementation of ASP and/or promote AS	
	Limited guidance from public authorities on initiatives to improve antibiotic use, ASP implementation, etc.	
	Coronavirus disease 2019–related exhaustion	Participation of quality improvement projects
	Social climate	Reporting antibiotic use to public authorities
	Economic prosperity	Benchmarking

Abbreviations: AS, antimicrobial stewardship; ASP, antimicrobial stewardship program; EMR, electronic medical record; IT, information technology.

Formal hospital leadership support for an ASP through a written document with designated individuals responsible for the ASP occurred in 50% of for-profit and only 17% of not-for-profit hospitals. Twenty-five percent of for-profit and 13% of not-for-profit hospitals reported meeting the minimum recommended AS physician-to-bed ratio (1 FTE [full time equivalent]/500 bed-size for AS activities) [9]. Similarly, 95% of hospitals reported inadequate pharmacy staffing (none of the for-profit hospitals and 8% of the not-for-profit hospitals reported 1 AS pharmacist FTE/500 bed ratio with 20% reporting no pharmacy support at all). Most participants reported a lack of dedicated time for AS, excessive workload with many physicians reporting working multiple jobs, and inadequate compensation for the type of work being performed.

“*I would describe it as partial support. There is a document that describes the goals of the ASP but we do not have dedicated time to perform AS activities. It is another activity we have to do on top of our clinical duties*” *(ID physician)*.

Participants perceived that lack of formal hospital leadership support was related to hospital leaders' limited understanding of the scope of the problem of AMR and the elements needed to implement an ASP or financial constraints preventing the allocation of resources for ASPs.

“*They don't think antimicrobial stewardship is a priority. When we told them the cost associated with a multi-drug resistant infection, only then, they became interested in supporting the ASP*” *(ID physician)*.

*“They [hospital leadership] don't understand we need a pharmacist in the team*” *(ID physician)*.

“*We need them [hospital leaders] to sit down with the relevant stakeholders and ask us what we need to do our job better*” *(Pharmacist)*.

Some hospitals reported high staff turnover across roles, which was mostly attributed to suboptimal salaries and work climate. Participants from not-for-profit hospitals also reported frequent turnover of hospital leadership (jobs might be driven by politics rather than merit), which was perceived as a barrier to sustainable ASPs.

Although 65% of hospitals reported having an AS committee, 80% indicated it was informal and without authority to implement or change policies, and 90% met fewer than 4 times per year. Participants perceived lack of time and overwhelming workload as major barriers to more frequent meetings. Some participants reported that building an ASP within the existing structure of the infection prevention and control (IPC) program was helpful. It was the participants' opinion that hospital leadership could be highly influential in promoting AS activities among HCWs and unit leaders (“top-down approach”).

”*We need the hospital director to set the expectations…. We need their support to communicate the need for certain practice changes” (ID physician)*.

**Behavioral determinants**

Most (63%) for-profit hospitals reported that patient safety surveys are regularly conducted in their facilities, while this occurred in only 17% of not-for-profit hospitals. Similarly, 88% of for-profit hospitals reported having an anonymous adverse event reporting system, while this was available in only 50% of not-for-profit hospitals. Implementation of strategies to promote a teamwork approach was uncommon (reported by 50% of for-profit and 25% of not-for-profit hospitals). Interviews revealed that hierarchical relationships remain a barrier to multidisciplinary work.

“*AS is centered too much around ID, with little participation of pharmacy or microbiology. I think we need to change that” (microbiologist)*.

*“Physicians have asked me who I think I am to tell them what to do with antimicrobials” (pharmacist)*.

Fear of loss of prescriber autonomy was apparent during interviews.

“*The hospital director prohibited prior authorization as he considered this to be a block to patient care” (ID physician)*.

**Education and training**

Education scored lowest on the self-assessment. During interviews, participants indicated limited opportunities for the AS team to train in AS. Hospitals do not usually provide paid time or financial support for training, forcing HCWs to pay out of pocket or accept industry funds for training during vacation time or after hours. Participants highlighted specific knowledge needs related to ASP implementation, including AS metrics and how to use these data to change behavior.

“*I want to learn about strategies to change behavior, and how to better communicate not only with peers but with other members of the medical team” (ID physician)*.

One hospital reported significant benefit from including an expert in health administration/patient safety on their team.

“*She (quality improvement expert) was very helpful in creating meaningful antibiotic use data reports, and in implementing a quality improvement initiative to improve antibiotic use at end of life.…She was very helpful in communicating the purpose of the initiative with physicians” (ID physician)*.

Participants also perceived that HCW's lack of knowledge of the local AMR or AS principles was an obstacle to improving antibiotic use.

“*They [physicians] just don't know it is not the same thing to give 14 days than 7 days of antibiotics” (Pharmacist)*.

*“Private physicians are not expected to follow the same rules as everyone else since they bring business to the hospital” (ID physician)*.

**Tasks**

As previously mentioned, most (95%) hospitals have inadequate pharmacy staffing, and most pharmacists have not received training in AS. During interviews, it became apparent that the role of pharmacists is generally limited to dispensing antimicrobials, performing validation of antimicrobial doses, and assisting in obtaining antibiotic consumption data (a manual process for many). Some participants reported that including pharmacists in ID/AS rounds favored more awareness about AS and the value of pharmacists on the medical team.

“*The weekly meetings with the pharmacist helped create more interest from people in antibiotic management” (ID physician)*.

There were differences between for-profit and not-for-profit hospitals regarding the implementation of AS activities (Table 2). For example, while more than 80% of hospitals reported implementing post-prescription review with feedback (PPRF), only 63% of for-profit and 33% of not-for-profit hospitals reported performing PPRF on a regular basis. Therapeutic drug monitoring for intravenous vancomycin or aminoglycosides was regularly performed in 63% of for-profit hospitals but only 17% of not-for-profit hospitals. Most hospitals (75%) reported implementing prior authorization for reserve antimicrobials in some areas/units of the hospital. During interviews, participants reported inefficiency in some of these processes.

“*The paper form is always incomplete, so I need to chase the physicians to get that information; otherwise, the therapeutics committee will not even consider it [restricted antimicrobial] for review and approval” (pharmacist)*.

Most hospitals reported monitoring antibiotic consumption and sharing (75% of not-for-profit and 62% of for-profit) antibiotic consumption data with other services/units in the hospital. Participants reported that antibiotic use data acquisition was very labor-intensive and time-consuming and that they lacked established venues to share these data with relevant stakeholders and to reach a wide range of individuals. For a few hospitals, obtaining aggregated data from microbiology (eg, antibiograms, rates of multidrug-resistant organisms) was a challenge (17% of not-for-profit hospitals were unable to obtain this information). Microbiologists reported staff shortages and work overload as the main barriers to having more participation in AS activities. Additionally, participants often mentioned inefficient communication between different members of the care team as a barrier to working more efficiently.

“*They call the lab instead of looking up the results in the computer, which we keep up to date. Answering those calls takes up a lot of our time” (microbiologist)*.

**Tools and IT**

Lack of syndrome-specific treatment guidelines was common (eg, 40% of hospitals did not have treatment guidelines for intraabdominal infections despite being a common driver of inpatient antibiotic use in Latin American hospitals [3, 10]). Participants perceived lack of time as the major barrier to writing or updating guidelines. One hospital took the initiative to task residents who rotate through the ID service to help with guideline writing.

“*Because we are a teaching hospital, a lot of the residents rotate in our services. I came up with the idea of asking residents to contribute to the ASP by reviewing literature and drafting or updating treatment guidelines” (ID physician)*.

Interviewees described inefficiencies related to guideline development and use.

“*We (ASP) have a few guidelines but so does internal medicine and surgery, and we all say something a bit different. We need to standardize guidelines, but no one has time to do it….” (ID physician)*.

Participants also reported behavioral barriers to better use of available guidelines.

“*We have our own guidelines that are based on our local microbiology; however, they prefer to use guidelines from another country because they think they are better than ours just from being from….” (pharmacist)*.

Most hospitals reported a lack of IT support (75% for-profit hospitals and 62% not-for-profit hospitals). During the interviews, participants identified several gaps related to IT including a lack of electronic prescriptions, which limits efficient tracking of antibiotic use for surveillance, and lack of efficient identification of antimicrobial orders for daily action, as well as limited availability of computers and lack of robust software to create microbiology reports (eg antibiograms).

“*We need to write down the susceptibilities on a piece of paper because the computers are far from the patients' charts where we write the antibiotic orders. That creates opportunities for errors” (ID physician)*.

*“The electronic prescriptions would save us so much time. I waste time walking around units looking for physician orders” (pharmacist)*.

**External environment**

Participants perceived external hospital accreditation, external audits by public health authorities, and participation in national or regional surveillance programs or research projects as opportunities that favor AS work. In our cohort, only a few for-profit hospitals currently are accredited or are seeking international hospital accreditation that, according to participants, helps hospital leadership become more aware of the importance of ASPs. Not-for-profit hospitals that can administer their budgets were more likely to report an environment that favors ASP implementation than not-for-profit hospitals that are almost exclusively dependent on central government budgets. Many participants highlighted the desire for more guidance and help from public health authorities regarding hospital ASP implementation.

“*The ministry asks us to submit hospital-acquired infections data. I think it would be helpful if we had to do the same for antibiotic use data” (ID physician)*.

Finally, the economic prosperity of the country was cited as a contributor to the suboptimal work climate, HCW exhaustion, and lack of engagement in AS activities.

We summarize future actions needed to implement effective ASPs in the region based on our study findings in Box 1.

Box 1: **Summary of future actions needed to implement robust and sustainable antimicrobial stewardship programs in Latin America.**

**Hospital Engagement and Support for ASPs**

- Identify the levers that will help hospitals create the necessary resources to support ASP implementation
- Set ASP as a hospital priority
- Improve engagement of hospital leadership and clinical leaders and directors in AS activities
- Ensure minimum staffing requirements, ensure ASP members have dedicated time to conduct an ASP

**AS Tasks**

- Identify key areas for improving antibiotic use locally
- Develop and disseminate guidelines for antibiotic use in conjunction with clinical leaders
- Improve processes for tracking and monitoring antibiotic use and resistance data
- Improve access to training related to antimicrobial stewardship for pharmacists, physicians, and nurses
- Improve information technology access to support AS activities (eg, for tracking data, identifying patients in whom to improve antibiotic use on)

**AS Culture**

- Engage and integrate frontline providers in AS activities (eg, share antibiotic use data, provide feedback on inappropriate use, engage them in improvement initiatives)
- Improve work climate and promote team work

**External Environment**

- Expand implementation resources for ASPs (eg, guidance on implementation of key strategies, educational resources about most common drivers of inappropriate antibiotic use)
- Develop methods by which antibiotic use/resistance data can be reported at a local or national level

## DISCUSSION

To our knowledge, we are the first to conduct an in-depth evaluation of barriers and facilitators of ASP implementation in Latin American hospitals through a self-assessment that evaluates ASP structure and activities and interviews those involved in AS activities who provided further insight as to the current barriers to ASP implementation.

We found inadequate technical and human resources to allow for the implementation of robust and sustainable ASPs. Additionally, cultural determinants remain an obstacle to the multidisciplinary work needed to improve antibiotic use in hospitals. Support from public health authorities continues to be limited.

Dedicated personnel are a basic pillar of any ASP. In our study, few hospitals met the minimum staffing recommendations for ASPs [9], with many participants reporting a lack of expertise in AS and quality improvement implementation. Participants reported excessive workload and multiple responsibilities often without the appropriate financial compensation. Similar to our findings, a recent global study that evaluated the implementation of IPC programs that included Latin American countries showed workload and staffing to be a major barrier to IPC program implementation [11]. Participants perceived financial constraints as a barrier to better resource allocation. Previous studies have shown that ASPs can be cost-effective in Latin American countries [12]; however, a better understanding of reimbursement models is needed to appropriately prioritize interventions to get ASPs started and to incentivize the development of ASPs in resource-limited settings [13]. Participants reported the need for cultural and organizational changes regarding how hospitals approach antibiotic use, with many citing power distance among HCWs (eg, physician–pharmacist, physician–nurse, surgeon–nonsurgeon), lack of cohesive work across disciplines (eg, redundant guidelines), and poor understanding of the consequences of inappropriate antibiotic use at the patient level. A recent national program aimed at strengthening AS across healthcare settings in the United States incorporated training in both technical (best practices for management of common syndromes) and behavioral (teamwork, effective communication) aspects of AS using patient safety as the framework to change antibiotic prescribing for patients across healthcare settings, which resulted in a significant reduction in antibiotic use and antibiotic-related adverse events [14, 15]. Collaboration with regional partners to build ASPs has been beneficial for resource-limited hospitals and might help mitigate the lack of trained dedicated personnel in some hospitals [16, 17]. Participants had very favorable views of public health activities related to AMR such as access to AMR testing and regional/national surveillance activities. However, they indicated the need for more robust support for training and guidance in ASP implementation but also in developing regulations to help prioritize ASP implementation and set minimum requirements for functioning programs.

Our study has some limitations. We used a convenience sample of hospitals and, as such, it does not represent an adequate sample of hospitals/population per country or for the region (Brazil, which is the most populous country in Latin America, was not included). We recruited hospitals through a regional network that was developed to help build ASPs, hence, these hospitals might be more engaged in ASPs and our results may underestimate barriers to ASP implementation. However, we identified similar gaps to prior studies that used different sampling methodologies [18, 19]. Our study did not specifically address how the coronavirus disease 2019 pandemic impacted ASP implementation in the region; however, participants mentioned both positive and negative consequences (eg, it increased specialty value and worsened clinical burden, respectively).

In summary, we identified common and unique barriers to the implementation of ASPs in for-profit and not-for-profit hospitals. We identified barriers with relatively easy solutions (eg, those related to education and training, increasing engagement from hospital administrators, and making a business case for ASP) and others that require longer-term efforts (eg, investments in IT resources, increasing access to clinical pharmacists, improved HCW compensation, less hierarchical relationships).

## Supplementary Data

Supplementary materials are available at *Clinical Infectious Diseases* online. Consisting of data provided by the authors to benefit the reader, the posted materials are not copyedited and are the sole responsibility of the authors, so questions or comments should be addressed to the corresponding author.

## Supplementary Material

ciad184_Supplementary_Data

## Notes

***Acknowledgments.*** This study was funded by the Centers for Disease Control and Prevention (CDC). The authors thank the healthcare workers who provided their time to participate in the interviews. They thank Guadalupe Reyes-Morales, Alejandra B. Salinas, and Romina Baez for their assistance with project coordination.

***Supplement sponsorship.*** This article appears as part of the supplement “The Evolving Challenges of Antibiotic Resistance in Low- and Middle-Income Countries: Priorities and Solutions,” sponsored by the U.S. Centers for Disease Control and Prevention, and Health Security Partners.

***Financial support.*** This article appears as part of the supplement “The Evolving Challenge of Antibiotic Resistance in Low- and Middle-Income Countries: Priorities and Solutions” sponsored by Health Security Partners.
