# Supplementary material for: Deep Dive Into Gaps and Barriers to Implementation of Antimicrobial Stewardship Programs in Hospitals in Latin America
Source: Clin Infect Dis. 2023 Jul 5;77(Suppl 1):S53–61. doi: 10.1093/cid/ciad184 (PMC10321692; doi:10.1093/cid/ciad184)
Supplement: ciad184_Supplementary_Data [file ciad184_supplementary_data.zip › Supplementary material_Fabre.docx]

**SUPPLEMENT****ARY MATERIAL**

## Interview Guide for Physician and Pharmacist Involved in Antimicrobial Stewardship Programs.

1. **Structure of the ASP**

Is there an ASP in your institution agreed and approved by hospital leadership (with objectives, strategies, managers, indicators, which is renewed annually)?

**If they say, "NO":** Despite not having a formal ASP, are activities carried out to optimize the use of antimicrobials in your institution?

1. How long have you been working on ASP or activities for the optimization of antimicrobials?

Is there a specific budget (economic resources allocated) for ASP or optimization activities? (Example: training course, paid protected time)

1. Is there an IPC in your institution?

Is the ASP team or ASP committee independent of the infection prevention and control team and committee?

**If they say YES:** Who is part of the ASP? (Roles, not names)

**If they say "NO":** Is anyone within the committee more in charge of the ASP?

*Ask if anyone and who in this committee is most in charge of ASP

1. What do you consider to be the objectives/main goals of ASP?
2. What would you need to achieve these objectives?
3. How would you describe the support from hospital leadership to the ASP? (or for activities for the optimization of antimicrobials?)
4. Is there a relationship between the Ministry of Health or local/regional government health organizations and ASP? Please describe what this relationship is like.
   1. How does ASP benefit from this relationship?

How much time would you say you spend on ASP activities per day or per week?

1. Could you describe a typical day at work as part of the ASP team?
2. **Communication, training, activities**

**Communication:**

1. How do you think the relationship between the members of the ASP team or between the members who carry out activities for the optimization of antimicrobials is?
2. How would you describe the working relationship and communication of the ASP with infectious disease doctors (who are not part of the ASP)? How would you describe the relationship of the ASP with the attending physicians and with the heads of unit?
   1. Do you think the relationship varies by service? why?
3. How would you describe ASP's collaboration with clinical pharmacists (who are not part of ASP)? (Ask them to give examples of cooperation activities)
4. How would you describe ASP's collaboration with the microbiology laboratory? (Ask them to give examples of cooperation activities)

**Training and support:**

1. What kind of training would you like to receive to continue developing ASP activities? In what topics?
2. What barriers do you have to train yourself (to participate in congresses, subscribe to journals), train health workers, and patients and families?

**Activities:**

1. How do you think the ASP is working in your institution? How do you think antimicrobial optimization activities are working?
2. **Facilitators & barriers**
3. Of these measures, which have you tried to implement and maintained?

- Adaptation of clinical treatment guides.
- Antimicrobial control strategies:
  1. Auto-stop
  2. Restriction of antimicrobials and dispensing with approval of someone
  3. Audit with feedback
  4. Daily real-time visit and discussion of treatments
  5. Validation by the pharmacist of the antimicrobial scheme in terms of dosage
- What do you think helped make this activity successful?
- How was the success of this ASP intervention measured?
- Were there any key actor(s) to facilitate the intervention?
- Why do you think it couldn't be implemented? (try to dig deeper than one why)
- Were there any key actor(s) that made the intervention difficult?
- Measurement of consumption and/or appropriability of antimicrobial use.
  1. Any other measures that you want to tell us?

1. What are the main difficulties in carrying out the objectives or priorities of the ASP? If there is no program, what difficulties have you encountered in organizing and starting an ASP?
2. **Antimicrobial Prescription**
   - 1. Generally speaking, what do you think is using antimicrobials in your hospital? (Excellent, very good, regular, bad, very bad) Why?
     2. Has this weighting been measured in any way or do you say so by observations in your daily practice?
     3. What are the main reasons that contribute to the inappropriate use of antimicrobials in your hospital? (Try to get something out of this question)
     4. What things would be a priority to improve in terms of use?

##

## Interview Guide for Microbiologists Involved in Antimicrobial Stewardship Programs.

1. **Structure and operation**
2. What is your role as a microbiologist in the ASP?
3. About antibiograms, for which areas of the hospital are made? If they do them by type of sample and / or by type of patient.
4. Is there a relationship between your institution's microbiology laboratory and the Ministry of Health or local government health organizations? Could you describe what this relationship looks like?
5. If there is any joint activity, what is the benefit to the laboratory?
6. If they report to the authorities, do they receive training?
7. Is there a return of data?
8. **Actors, Resources and Activities**
9. How would you describe the collaboration with ASP?
   1. Ask them to describe how they collaborate.
10. How is the relationship with infectious disease doctors (who are not part of the ASP)?
11. How is the relationship with healthcare doctors?
    1. Do you think the relationship varies by service? why?
12. **Resources**
13. Do you have the necessary resources to properly identify microorganisms and their mechanisms of resistance?
14. In general, how often do they run out of supplies to make microbiological diagnosis?
    1. How do they solve it?
15. Do you have rapid diagnostic techniques? Ex: Multiple PCR, MALDI-TOF

**If they say "YES":** Do you use that diagnostic test for all patients?

**If they say "NO”.** Ask why.

1. How do you have access patient clinical data to give context to culture results?
2. If a highly resistant bacterium is detected, to whom it communicates the results (they can be all):
   1. Treating team
   2. ASP
   3. Infection Control and Prevention Team
   4. National authorities

**Specific microbiological tests:**

1. How long to report the following to the care team?
2. the gram stain
3. the report of the name of the bacterium (typing)
4. the sensitivity report (which antibiotic you can use)
5. How do you handle the issue of blood cultures once the laboratory closes? (where do they store them, process them?)
6. Can you briefly describe the process of communicating results to the care team?
7. Can you mention what strategies have been implemented or are considering to detect colonization of the following organisms?:
   1. MRSA
   2. VRE
   3. ESBLs
   4. CRO
8. Can we talk about *Clostridioides difficile*? What are the diagnostic methods available in your institution?
9. What kind of respiratory samples do you process for the diagnosis of ventilator associated pneumonia? (Do you use techniques to differentiate contamination/low-quality sample?) Have you implemented any changes to the report to help treating physicians make more appropriate antibiotic decisions?
10. How about urine cultures? Do you have any strategy to report the result of a urine culture if the flora is mixed or is there is a low count of bacteria?
11. Let’s talk about susceptibility reports. Have you implemented any strategies that help the prescriber in the decision of antimicrobials?
12. Example: Suppress certain antibiotics
13. Example: Suppress reporting yeasts in respiratory samples.
14. **Facilitators & barriers**
15. What could the microbiology lab do to help optimize the use of antimicrobials at your institution?
16. What are the main difficulties that the microbiology laboratory has in supporting ASP and/or improving the use of antimicrobials in your hospital?
17. What could information and technology area do to collaborate with the microbiology laboratory to optimize the use of antimicrobials? (Implementation of a laboratory system)
18. What could the hospital leadership do to support the microbiology laboratory?

# Supplementary figure: Scores on the asp self-assessment by hospital type and sub-domains.
